# The Institutional Worker

**Published:** 1911-10-28

**Authors:** 


THE
### INSTITUTIONAL WORKER
**Being a Special Supplement to "The Hospital"**

### Editor's Notices.

**Contributions:**

Contributions should be written, or preferably typed, on one side of the paper only, and all articles sent in are accepted upon the distinct understanding that they are forwarded to The Hospital only.

The Editor cannot undertake to return MSS. not used, but when a stamped directed envelope is enclosed the MSS. may be returned if a special request is made.

Accepted articles and paragraphs of news will be paid for after publication at the scale rate.

**Address:**

To prevent delay all contributions and letters on editorial business must be addressed exclusively to the Editor, "The Hospital," 29 Southampton Street, Strand, London, W.C. It is important that this regulation shall be strictly observed.

**Correspondence:**

Correspondence on all subjects is invited. The name and address of correspondents must be given as a guarantee of good faith, but not necessarily for publication.

**Special Articles:**

Special articles are invited, and questions, inquiries, and paragraphs upon all matters relating to the work, administration, and management of general, special, mental, fever, cottage and convalescent hospitals, sanatoria, homes, institutions, societies and organisations for the treatment or care of the sick, injured and dependents of all classes. Every matter affecting the interests and welfare of the staff of all grades working in these institutions will receive special consideration.

**Administrative Medicine, Research and Items of News:**

Special terms will be paid for approved articles on subjects in this wide field by experts who have made some section of it their special study and interest. We wish to give prominence to every movement tending to advance accuracy of diagnosis, efficiency in treatment, and the development of modern methods to eradicate disease and promote the welfare of the suffering.

**A Bureau of Information:**

We invite inquiries and applications for information and help in providing for that numerous class of sufferers who require special care or house-room which they cannot obtain in their own homes or secure for themselves. **We cannot, however, prescribe or recommend practitioners.**

**Books for Review:**

Publishers are particularly requested to send advance proofs of any new books of importance whenever possible, as the Editor has made arrangements to publish immediate reviews on a new plan.

**Photographs, Plans, Blocks and Illustrations:**

It is requested that wherever possible MSS. may be accompanied by illustrations in any of the above forms. The name of the sender and of the article to which it belongs should be written on the back of each photograph, drawing, or block for purposes of identification.

**Local Papers:**

Newspapers containing reports or news paragraphs should be marked and addressed to the Sub-Editor.

**The Coupon System:**

A coupon will be found at the bottom of the third inside page of the cover each week. This coupon must be attached to every question or inquiry to which an answer is desired in The Hospital.

### THE FLORENCE NIGHTINGALE MEMORIAL AND HOSPITAL COMMITTEES.

*To the Editor of* The Hospital.

The Honorary Secretary to a Provincial Hospital writes: It will be difficult to raise even a moderate sum, though it be for so worthy an object. However, I will see what can be done. The fact also remains, the reverence and respect felt for Florence Nightingale by the older generation of hospital workers does not impress itself on the modern workers. To them she has become "ancient history." Few of them could tell you actually what she did; fewer still could follow the movement she initiated and could point out the far-reaching influence which has made the modern hospital what it is. Let a second year pass away, and the ordinary hospital committeeman, if her name is mentioned in his presence, will say: "Oh, ah! I have heard that name before; now, who was she?" I will let you know what success I meet with.

[In thanking "Honorary Secretary" for his kind co-operation, we feel moved to invite correspondence on the point this letter raises. Can the knowledge and interest of the average hospital committeeman be, in fact, as little as is here represented?—Ed. The Hospital.]

### Manager's Notices.

Letters relating to the publication, sale, and advertising departments of The Hospital must be addressed to the Manager, "The Hospital," The Hospital Building, 28 and 29 Southampton Street, Strand, London, W.C.

**Circulation and Sale:**

The Hospital is on sale at all newsagents and bookstalls every Friday, price one penny. The rates of subscriptions, post free from The Hospital office, have been reduced and are now as follows :—

| | United Kingdom s. d. | The Colonies and Abroad s. d. |
|---|---|---|
| For one year ... ... ... | 6 6 | 8 8 |
| ,, six months ... ... | 4 0 | 5 0 |
| ,, three months ... ... | 2 0 | 2 6 |

**Subscriptions**, which may begin at any time, are payable in advance. Cheques and Post-Office orders, crossed London County and Westminster Bank, Covent Garden branch, should be made payable to the Manager of The Hospital and addressed to him at The Hospital Building, 28 and 29 Southampton Street, Strand, London, W.C.

**Advertisements:**

All advertisements for The Hospital must reach the Manager not later than Wednesday morning in each week. For scale of charges see pages v and 2.

**Special Rates** will be quoted for those institutions which agree to send all their vacancy, contract, and public notices to The Hospital each year, so as to make it a file of such announcements for ready reference.

**Classified Advertisements:**

All small advertisements will be classified, for this section of the paper is widely read and of special interest. We wish to encourage those wanting appointments who are attracted to institutional work, and therefore offer them the reduced rate of *twenty-one words and under for* 1s., and *for every ten and part of ten words beyond*, 6d.

### APPOINTMENTS VACANT.

### Poor-Law Vacancies.

### Appointments Vacant.

Assistant Master (£40), Woolwich Union. Mr. T. Cutter, C. to G., Woolwich. Nov. 2.

Assistant Nurse (£20), Dover Union. Mr. E. Carder, C. to G., Dover. Oct. 31.

Assistant Nurse (£26), Lincoln Union. W. B. Danby, C. to G., Workhouse, Lincoln. Oct. 30.

Assistant Nurse (£30), West London School District. F. G. Beeching, Clerk West London Schools, Ashford, Middlesex. Oct. 30.

Bandmaster (£65), Strand Union. Mr. A. H. Maddocks, C. to G., 15 Henrietta Street, Covent Garden, W.C. Nov. 1.

Charge Nurse (£28), Brighton Guardians. B. Burfield, C. to G., Parochial Offices, Brighton, Nov. 6.

Charge Nurse (£24), City of London Workhouse and Infirmary. E. R. Woodward, C. to G., 42 Clifden Road, Median Road, N.E.

Charge Nurses (2) (£30), Dewsbury Union. Mr. C. P. Pickersgill, C. to G., Dewsbury. Nov. 1.

Female General Assistant (£30), Risbridge Union. Mr. S. L. Bigmore, C. to G., Haverhill, Suffolk. Nov. 10.

Female Storekeeper (£25), Salford Union. Mr. F. Townson, C. to G., Salford. Oct. 31.

Foster-Mother (£25), Bucklow Union. Mr. G. Leigh, C. to G., Knutsford. Nov. 6.

Foster-Mother (£20), Grimsby Union. Mr. J. F. Wintringham, C. to G., Grimsby. Oct. 31.

Foster-Mother (£25), Relief Foster-Mother (£18), Canterbury Guardians. Mr. J. Plummer, C. to G., Canterbury. Nov. 6.

Foster-Mothers (£25 and £20), Rochford Union. Mr. F. Gregson, C. to G., Southend-on-Sea. Oct. 30.

Foster-Mother (£30), St. Alban's Union. Mr. R. W. Brabant, C. to G., St. Albans. Oct. 28.

Foster-Mother (£24), Steyning Union. Mr. A. Flowers, C. to G., Shoreham-by-Sea. Oct. 30.

Head Nurse (£30), Holbeach Union. S. S. Mossop, Jun., C. to G., Holbeach. Oct. 28.

Infirmary Matron and Superintendent Nurse (£80), Wigan Union. Mr. H. G. Ackerey, C. to G., Wigan. Nov. 2.

Labour Mistress and Assistant Needlewoman (£25), Reigate Guardians. Mr. F. C. Morrison, C. to G., Reigate. Nov. 2.

Male Attendant (£30), Poplar Guardians. Mr. G. H. Lough, C. to G., Upper North Street, Poplar, E. Oct. 30.

Massage Sister (£50), Fulham Guardians. Mr. E. J. Mott, C. to G., 129 Fulham Palace Road, W. Oct. 31.

Master and Matron (£70 and £40), Chelmsford Union. Mr. A. S. Duffield, C. to G., Chelmsford. Nov. 1.

Master and Matron (£60 and £40), Lancaster Union. Mr. W. D. Ball, C. to G., Lancaster. Nov. 13.

Master and Matron, West Ham Union. Mr. T. Smith, C. to G., Leytonstone, N.E. Nov. 9.

Master's Clerk and Storekeeper (£35), Medway Union. Mr. A. R. Norman, C. to G., Chatham. Nov. 3.

Midwife (£30), Southwark Union. Mr. S. Wood, C. to G., Union Offices, Ufford Street, Blackfriars, S.E. Oct. 31.

Nurse (£40), Cavan Union. P. Reilly, C. to G., Union Offices, Cavan. Nov. 6.

Nurse (£25), County Wexford Infirmary. M. J. Kavanagh, Registrar. Nov. 6.

Nurse (£30), Manchester Guardians. Matron of the Schools, Swinton, Manchester. Oct. 28.

Nurse (£30), Medway Union. A. Reynolds Norman, C. to G., 22 High Street, Chatham. Nov. 10.

Nurse (£20), St. Pancras Guardians. J. E. P. Hall, C. to G., Town Hall, Pancras Road, N.W.

Nurse (£30), Wellington Union. R. Gwynne, C. to G., Edgbaston House, Wellington, Salop. Nov. 9.

Nurses (2) (£30 each), Winchester Guardians. Mr. F. Faithfull, C. to G., Winchester. Oct. 30.

Probationer Nurses (£10), Medway Union. A. Reynolds Norman, C. to G., 22 High Street, Chatham.

Probationer Nurse (£12), Uxbridge Union. C. Woodbridge, C. to G., 38 High Street, Uxbridge. Oct. 28.

Relieving Officer, Etc. (£102 and commission), Erpingham Union. J. T. Willis, Deputy C. to G., Cromer Road, Sheringham. Oct. 28.

Superintendent Nurse (£40), Newbury Union. S. V. Penniger, C. to G., Market Place, Newbury. Nov. 3.

Superintendent Nurse (£45), Isle of Thanet Union. C. Taylor, C. to G., Board Room, Minster, nr. Ramsgate. Nov. 16.

Superintendent Nurse (£35), Wisbech Union. Mr. T. H. Stockdale, C. to G., Wisbech. Oct. 30.

Superintendent of Male Casuals (£30), Brighton Guardians. Mr. B. Burfield, C. to G., Brighton. Oct. 31.

Temporary Charge Nurse (£40), Brighton Guardians. B. Burfield, C. to G., Parochial Offices, Brighton. Nov. 6.

Valuer, Blean Union. Mr. J. E. Burch, C. to G., 39 Castle Street, Canterbury. Oct. 30.

Ward Sister (£30), Southampton Union. A. J. Walden, C. to G., Workhouse, Southampton. Oct. 28.

Ward Sister (£32), Prestwich Union. E. W. Ogden, C. to G., Cheetham Hill Road, Manchester. Oct. 30.

### Situations Vacant.

Cook (£30), West London School District. Mr. F. G. Beeching, Clerk to the Managers, Ashford, Middlesex. Oct. 30.

Dispensary Porter (9s. weekly), Hammersmith Guardians. The C. to G., 206 Goldhawk Road, Shepherd's Bush, W. Oct. 30.

Female Cook (£30), Bucklow Union. Mr. G. Leigh, C. to G., Knutsford. Nov. 6.

Female Cook (£25), Totnes Union. Mr. F. K. Windlatt, C. to G., Totnes. Nov. 2.

Laundress (£30), Bucklow Union. Mr. G. Leigh, C. to G., Knutsford. Nov. 6.

Laundress (£28), Darlington Union. Mr. C. H. Leach, C. to G., Darlington. Oct. 28.

Porter (£26), Salford Union. Mr. F. Townson, C. to G., Salford. Oct. 31.

Porter and Labour Master and Porteress (married couple) (£35 and £25), Kingston-upon-Hull Guardians. Mr. R. H. Winter, C. to G., Hull. Oct. 31.

### Institutional and Administrative Appointments.

Matron (£70), Hospital for Women, Nottingham. Apply Secretary. Oct. 31.

Probationers, Royal Sussex County Hospital. Apply Matron.

Staff Nurse (£24), Bangor (County Down) Infirmary. Apply Matron. Nov. 1.

### Municipal Vacancies.

### Appointments Vacant.

Assistant Nurse (£25), Newton Abbot Isolation Hospital. J. Underhill, Clerk to the Hospital Committee, Newton Abbot. Nov. 7.

Assistant Nurse (£20), Stafford Isolation Hospital. The Town Clerk, Stafford. Oct. 28.

Assistant Ophthalmic Nurse (30s. weekly), Manchester Town Council. Mr. T. Hudson, Town Clerk, Manchester. Oct. 28.

Assistant Sanitary Inspector (£78), Burnley T.C. Mr. P. Thomas, Town Clerk, Burnley. Oct. 30.

Assistant Surveyor (£65), Rothwell U.D.C. Mr. W. Dodgson, Clerk to the Council, Rothwell. Nov. 2.

Borough Engineer (£450), Bury T.C. Mr. J. Haslam, Town Clerk, Bury. Oct. 31.

Borough Treasurer and Accountant (£500), Sunderland T.C. Mr. F. M. Bowey, Town Clerk, Sunderland. Oct. 31.

Bridge and Road Surveyor (£400), Devon C.C. Mr. H. Michelmore, Clerk to the Council, Exeter. Oct. 30.

Clerk of the Peace (£755), Pembroke Standing Joint Committee. M. George, Shire Hall, Haverfordwest. Nov. 3.

Draughtsman and Analyst for Gas Department (£100); Huddersfield T.C. Mr. J. H. Field, Town Clerk, Huddersfield. Oct. 28.

Engineering Assistant (£130), Hackney B.C. Mr. W. A. Williams, Town Clerk, Hackney. Oct. 31.

Gas Engineer and Manager (£1,000), Nottingham T.C. J. A. H. Green, Town Clerk, Nottingham. Nov. 2.

Health Visitor (£80), Kirkby-in-Ashfield U.D. Council. S. B. Hibbert, Clerk, 45 Westgate, Mansfield, Notts. Oct. 28.

Health Visitor (£105), Borough of Bermondsey, F. Ryall, Town Clerk, Bermondsey. Oct. 31.

Junior Accounts Clerk (£75), Chislehurst U.D.C. Mr. H. E. Knight, Clerk to the Council, Chislehurst. Nov. 1.

Matron (£80), Brighton County Borough Asylum. H. Talbot, Town Hall, Brighton. Nov. 6.

Nurse (£35), Brandon and Byshottles U.D. Council. A. A. Luxmoore, 5 North Bailey, Durham. Nov. 13.

Nurse £80), Cavendish Road School, Balham. L. Gomme, Clerk of the London County Council, Education Office, Victoria Embankment, W.C. Nov. 4.

Nurse (£25), Pontypridd Isolation Hospital. Medical Officer of Health, Pontypridd.

Second Assistant Clerk (£90), Maldens and Coombe U.D.C. Mr. J. W. Johnson, Clerk to the Council, New Malden. Oct. 28.

School Attendance Officer (£85), Heston and Isleworth U.D.C. Education Committee. Mr. M. Armstrong, Secretary to the Committee, Hounslow. Nov. 1.

School Nurses (£75), Swansea Education Committee, A. W. Halden, Clerk, Education Offices, 9 Grove Place, Swansea. Nov. 1.

Sister (£33), Rothwell Isolation Hospital. W. B. Pindar, Leek Street, Hunslet, Leeds. Nov. 4.

Superintendent of Cemetery (£75), Watford Burial Board. T. J. Broad, Clerk to the Board, Watford. Oct. 31.

Temporary Nurses (£24), Croydon Rural and Merton Joint Hospital Board. E. J. Gowen, Clerk, Council Offices, Katherine Street, Croydon.

Third Invoice Clerk (£78), Islington B.C. Mr. W. F. Dewey, Town Clerk, Upper Street, N. Nov. 1.

### Situations Vacant.

Town Hall Keeper (£26 and residence), Wokingham T.C. Mr. J. May, Town Clerk, Wokingham. Oct. 28.

### Miscellaneous Vacancies.

### Situations Vacant.

Head Laundress (£30), Berry Wood Asylum, Northampton. Apply Medical Superintendent.

School Nurse (£70), Walthamstow Education Committee. T. W. Liddiard, Secretary, High Street, Walthamstow. Nov. 6.

